# Healthcare professionals’ knowledge and attitudes toward the microbiome and fecal microbiotherapy or microbiota transfer in hematology and oncology: an international survey

**DOI:** 10.46989/001c.166241

**Published:** 2026-09-16

**Authors:** Marion Larue, Giorgia Battipaglia, Hendrik Poeck, Riccardo Masetti, Christoph Stein-Thoeringer, Jaime Sanz, Daniela Weber, Annalisa Ruggeri, Zinaida Peric, Raphael Duarte, Mohamad Mohty, Florent Malard

**Affiliations:** 1 Sorbonne Université; Centre de Recherche Saint-Antoine INSERM UMRs938; Service d’Hématologie Clinique et de Thérapie Cellulaire, Hôpital Saint Antoine, AP-HP, Paris, France; 2 Hematology Department, Federico II University, Naples, Italy; 3 Department of Internal Medicine III, Hematology & Internal Oncology, University Hospital Regensburg, Regensburg, Germany; 4 Leibniz Institute for Immunotherapy (LIT), Regensburg, Germany; 5 Bavarian Cancer Research Center (BZKF), Regensburg, Germany; 6 Pediatric Unit Azienda Ospedaliero-Universitaria di Bologna; 7 Department of Internal Medicine I, University Hospital Tuebingen, Tuebingen, Germany; 8 Adult Bone Marrow Transplant Service, Division of Hematologic Malignancies, Memorial Sloan Kettering Cancer Center, New York, NY, USA; 9 Departament de Medicina, Universitat de Valencia, Valencia, Spain; 10 Department of Internal Medicine III, Ulm University Hospital, Ulm, Germany; 11 Hematology and Bone marrow Transplantation Unit, San Raffaele Scientific Institute, Milan, Italy; 12 University Hospital Centre Rijeka and School of Medicine, University of Rijeka, Rijeka, Croatia; 13 Department of Hematology, Hospital Universitario Puerta de Hierro Majadahonda, Madrid, Spain; 14 Sorbonne Université ; Centre de Recherche Saint-Antoine INSERM UMRs938; Service d’Hématologie Clinique et de Thérapie Cellulaire, Hôpital Saint Antoine, AP-HP, Paris, France

**Keywords:** FMT, Fecal Microbiotherapy, Microbiome

## Abstract

The gut microbiome has emerged as a critical factor influencing cancer progression, treatment response, and complications in hematology and oncology. Fecal microbiota transfer (FMT) is being explored as a novel biotherapeutic strategy, yet the knowledge and attitudes of healthcare professionals towards its clinical use remain poorly defined. We conducted an international survey of 152 healthcare professionals, including hematologists, oncologists, transplant specialists, and others. Most respondents were affiliated with academic centers (66.0%) and practiced primarily in hematology (48.0%) or transplantation (34.0%). Awareness of the microbiome’s clinical relevance was widespread, but perceptions of microbiome profiling and FMT remained cautious. One-third of participants were in favor of routine microbiome profiling, while half considered it promising but still investigational. Direct clinical experience with FMT was limited: only 20.4% had prescribed it and 14.5% had referred patients. Nonetheless, over half indicated they would consider adopting FMT in the near future. The main reported barriers to broader use included limited regulatory approval (63.8%), logistical or supply challenges (57.9%), and physician skepticism or lack of knowledge (51.3%). Despite these challenges, more than 70% of respondents expressed strong interest in further education, standardized guidelines, and training opportunities to support safe and effective implementation. This survey highlights a strong enthusiasm regarding microbiome-based interventions in cancer and transplantation care. While current clinical use remains limited, healthcare professionals recognize the therapeutic potential of FMT and call for structured education, multidisciplinary collaboration, and clear regulatory frameworks to enable its integration into routine practice.

## INTRODUCTION

The human gut microbiome is increasingly recognized as a critical regulator of host immunity, metabolism and treatment outcomes in cancer care.^1^ Advent of sequencing technologies have revealed strong links between the composition of gut microbes and host cells, thus and responses to therapy in hematology and oncology.^2,3^ Disruption to intestinal microbial diversity, which is often induced by chemotherapy, antibiotics or haematopoietic cell transplantation (HCT), has been linked to infectious complications, immune dysregulation, metabolic imbalance and reduced survival.^4–6^ Loss of microbial diversity in particular has been associated with a higher incidence and severity of graft-*versus*-host disease (GVHD), as well as increased non-relapse mortality.^7,8^ In addition, use of broad-spectrum antibiotics and gut microbiota dysbiosis in the weeks before CD19-CAR-T cell infusions has been demonstrated to be associated with significantly reduced overall survival and progression or relapse of the malignant disease.^3,9,10^

Fecal microbiota transfer (FMT) is a strategy that restores the balance of microbes in the gut by transferring microbiota from healthy donors.^11,12^ It has demonstrated remarkable efficacy in treating recurrent *Clostridioides difficile* infection.^13,14^ Encouraged by these results, FMT is now being investigated in oncology, HCT and cellular therapy.^12,15–17^ Early clinical studies suggest potential benefits in managing steroid-refractory GVHD, reducing post-transplant infections, and enhancing responses to immune checkpoint inhibitors.^18,19^ Nevertheless, FMT remains investigational in hematology and oncology, with limited implementation outside of clinical trials due to regulatory, safety and logistical concerns.^12,20^

Healthcare professionals play a pivotal role in adopting novel therapeutic strategies. Their knowledge and perceptions are crucial in bridging the gap between emerging microbiome science and patient care. However, little is known about the current awareness of and adoption towards microbiome-based interventions among oncologists, hematologists, and HCT specialists. It is crucial to identify existing knowledge gaps and perceived barriers in order to guide educational initiatives and develop consensus guidelines that will support the safe and effective implementation of these interventions.

This international survey was therefore designed to assess healthcare professionals’ knowledge, adoption and practices regarding the microbiome and FMT in hematology and oncology. By providing insights into current perceptions, the study will inform future strategies for integrating microbiome-based interventions into standard cancer and HCT care.

## MATERIALS AND METHODS

All members of the International Academy for Clinical Hematology (IACH; www.iach.org) with an interest in allogeneic HCT and GVHD were invited to participate in this survey in April 2025. A questionnaire was distributed, followed by two reminders. The questionnaire included the following sections: demographic and professional background, self-reported knowledge of the microbiome, awareness and perceptions of FMT and other microbiome-targeted interventions, attitudes toward microbiome profiling, educational needs and barriers to implementation. Responses were collected anonymously. Descriptive statistics were used to analyze data and are expressed as frequencies and percentages.

## RESULTS

A total of 152 responses were received from approximately 1,000 invited healthcare professionals (response rate approximately 15%). Respondents originated from more than 40 countries across Europe, Asia, Africa, and the Americas. The demographic and professional characteristics of the participants are summarized in **Table 1**.

**Table 1.** Demographic and professional characteristics of survey respondents (n = 152)

**Characteristic**	**n (%)**
***Practice setting***	
– Academic hospital / University medical center	100 (66.0)
– Public hospital	29 (19.3)
– Private hospital	9 (6.0)
– Research institute	8 (5.3)
– Other	6 (3.9)
***Primary specialty***	
– Hematology	73 (48.0)
– Hematopoietic cell transplantation	52 (34.0)
– Medical oncology	8 (5.3)
– Infectious diseases	4 (2.7)
– Gastroenterology	3 (2.0)
– Clinical microbiology	2 (1.3)
– Other	10 (6.7)
***Countries with the highest participation***	
– France	12 (8.1)
– Turkey	11 (7.4)
– Italy	8 (5.4)
– Spain	6 (4.1)
– Greece	5 (3.4)
– India	5 (3.4)
– United Kingdom	5 (3.4)
– United States	5 (3.4)

Countries represented by fewer than five respondents included Germany (n = 4), Egypt (n = 4), Romania (n = 4), Serbia (n = 4), Canada (n = 3), China (n = 3), South Africa (n = 3), Tunisia (n = 3), and more than 30 additional countries.

### Institutional and professional background

The majority of respondents were affiliated with academic hospitals/university centers, followed by public hospitals, private hospitals, and research institutes. Almost half of the participants reported hematology as their main area of clinical practice, while one-third specialized in HCT.

### Perceived importance of FMT

Most respondents considered FMT highly relevant to improving outcomes in hematology and oncology. The majority rated it as very important (65.8%) or moderately important (28.3%). Only a small minority regarded it as minimally important (3.3%) or were unsure (2.6%). None of the respondents considered it unimportant.

Opinions varied regarding the most appropriate time for introducing FMT into cancer or haematopoietic cell transplant care. The most frequent choice was curative or adjuvant use in combination with other established therapies (37.5%). Preventive use, such as before/after antibiotics or high-dose chemotherapy, was endorsed by 32.9% of respondents. Early intervention, based on detection of microbiome disruption, was supported by 15.8%, while 6.6% believed it should be reserved for use only after standard therapies have failed. A small proportion (7.2%) were uncertain.

### Clinical experience with FMT

Direct clinical experience with FMT was limited (**Figure 1A**). Clinical experience referred to any prior use of FMT, including participation in clinical trials, compassionate use programs, or routine clinical practice according to local regulations.

**Figure 1.**
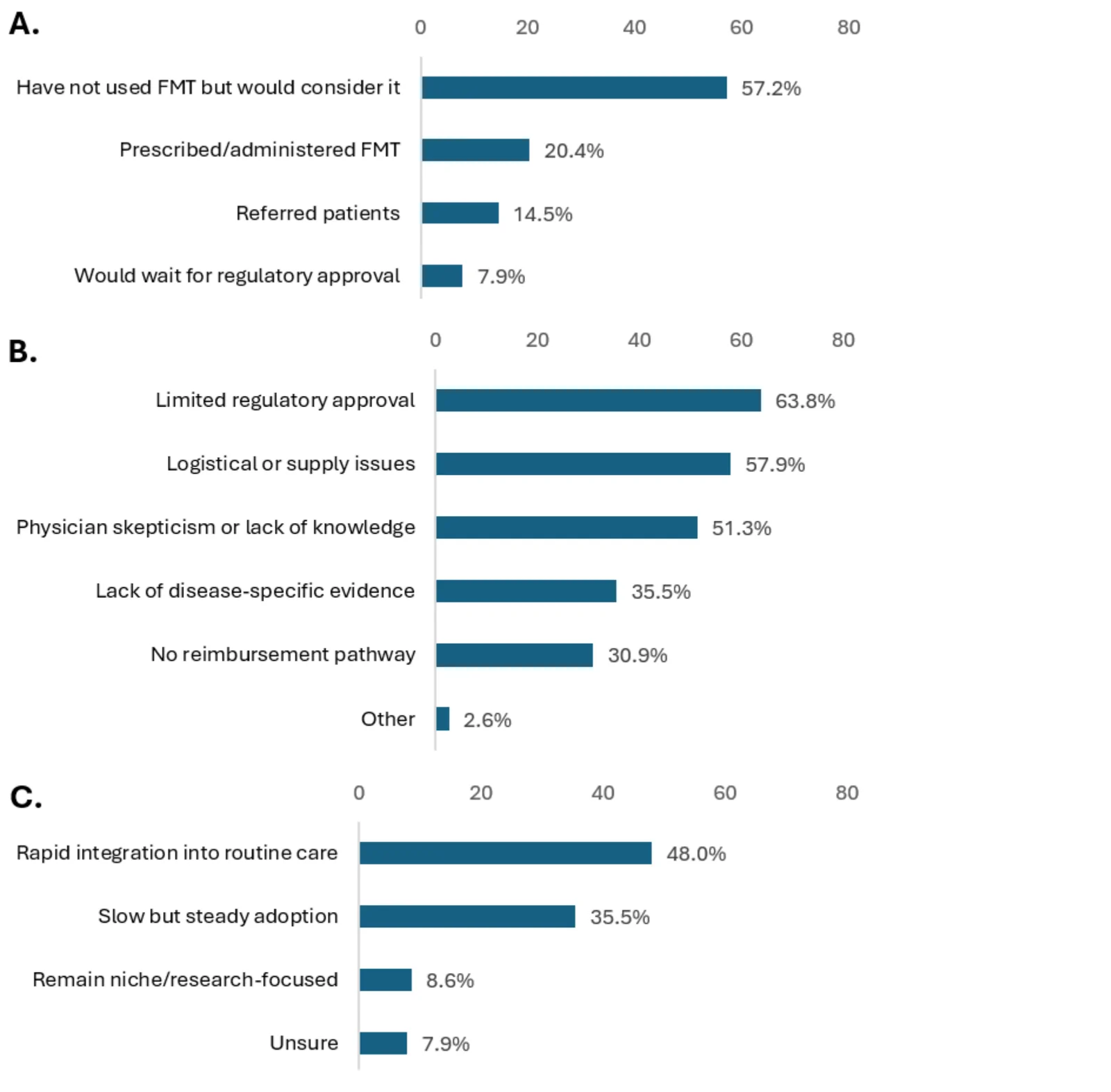
Survey results illustrating healthcare professionals’ clinical experience (**A**), perceived barriers to implementation (**B**), and future perspectives (**C**) regarding fecal microbiota transplantation (FMT) in hematology and oncology.

When considering both actual and potential clinical uses of FMT among all respondents, the most frequently cited were GVHD (65.8%), and recurrent *Clostridioides difficile* infection (58.6%). Other reported contexts included multi-drug-resistant bacteria eradication (46.1%), cancer immunotherapy-related colitis (30.3%), neutropenic enterocolitis (25.7%), and resistance to immune checkpoint inhibitors (17.8%). A smaller proportion did not indicate the indication (7.9%) or mentioned other conditions (2.0%).

Awareness of existing clinical guidelines or position statements regarding FMT in oncology and hematology was generally limited. More than half of the respondents (53.9%) reported being unaware of any guidelines. A smaller proportion indicated awareness without having applied them clinically (23.7%), while 17.1% stated that they had already used such guidelines in practice. A minority (5.3%) were uncertain. These findings suggest that although some clinicians have begun to reference or implement guidance on FMT, the majority do not currently identify established recommendations in their clinical practice environment.

### Perceptions of FMT: patients versus physicians

From the patient perspective, as reported by clinicians who already used FMT, acceptance of FMT was generally favorable but not universal. About one-third (32.9%) of respondents indicated that most patients are open to receiving the treatment when it is thoroughly explained. Another 30.9% had mixed reactions, with some hesitance remaining. A smaller group (5.3%) reported frequent resistance among patients. From the physician perspective, the main concern was the absence of clinically demonstrated safety profile of the intervention. A total of 37.5% considered FMT safe, based on published data, and 34.9% rated it as acceptable though with potential risks. However, 16.5% stated that the safety remained unclear until supported by additional clinical trials, and 11.2% reported no opinion.

Taken together, these findings suggest that while patients can often be reassured through appropriate counseling, physicians themselves retain a degree of caution, primarily regarding safety and the strength of supporting evidence. This dual perspective underscores the importance of both patient education and further clinical validation to promote broader adoption of FMT in oncology and hematology.

### Barriers to clinical adoption

In parallel, several obstacles were identified as limiting the broader clinical use of FMT in oncology and hematology (**Figure 1B**). These results highlight that beyond scientific and clinical uncertainties, regulatory, logistical, and educational challenges remain major impediments to the integration of FMT into standard hematology and oncology practice.

### Future perspectives

Expectations for the next five years were largely optimistic (**Figure 1C**). Finally, when asked about the regulatory classification of full-ecosystem microbiotherapies, over one-third of respondents viewed them as biological medicinal products, one-third (33%) favored classification as industrial-grade pharmaceutical products under GMP, and 16% supported the creation of a new regulatory category for microbiome-based therapies.

## DISCUSSION

This international survey provides new insights into healthcare professionals’ knowledge and practices regarding the microbiome and FMT in hematology and oncology. First, while recognition of the microbiome’s potential role is widespread, most participants considered microbiome profiling as promising but not yet standard-of-care. This cautious stance reflects the current status of microbiome science, where translational evidence is strong but prospective; randomized clinical trials are still needed to support routine clinical use.^16,20^

Secondly, the uptake of FMT in clinical practice remains low, with fewer than one-quarter of respondents having direct experience of it. However, the majority expressed willingness to adopt it as soon as evidence and regulatory approval become available. This emphasizes both the enthusiasm and the existing barriers, particularly regulatory, logistical, and educational.

Thirdly, a significant proportion of respondents identified educational gaps, expressing a lack of confidence in their ability to explain microbiome-based interventions to patients. This finding emphasizes the need for structured training programs and consensus guidelines, ideally developed by international societies, to ensure the safe and effective implementation of these interventions. Finally, the survey emphasizes the importance of multidisciplinary collaboration, given that microbiome interventions overlap with the fields of hematology, oncology, infectious diseases, gastroenterology and microbiology. Such collaboration is essential for designing protocols, standardizing procedures and ensuring patient safety.

Our study has several limitations. First, the response rate was relatively low (approximately 15%), which may have introduced selection bias. Healthcare professionals with a particular interest in the gut microbiome or FMT were likely more inclined to participate, potentially leading to an overestimation of awareness and enthusiasm regarding microbiome-based interventions. Second, respondents originated predominantly from academic and specialized centers, limiting the generalizability of our findings to other healthcare settings. In addition, the limited number of respondents outside Europe precluded a meaningful regional comparison, although regulatory frameworks and access to FMT vary considerably across countries and regions. Future international surveys with broader and more balanced geographical representation should specifically address these regional differences. Finally, the attitudes reported in this survey may not fully reflect actual clinical practice.This survey reveals enthusiasm regarding microbiome profiling FMT in hematology and oncology. Despite limited clinical experience, most professionals recognize the therapeutic potential of microbiome interventions. However, significant barriers remain, including lack of regulatory approval, standardization, and clinician training. Educational initiatives, multidisciplinary collaboration, and robust clinical trials are urgently needed to enable the safe and effective integration of microbiome-based therapies into cancer and transplantation care.

### AUTHOR CONTRIBUTIONS

Conceptualization: M.M., F.M., and M.L.; Data curation: all authors; Formal analysis: M.M. and M.L.; Writing – original draft: M.M. and M.L.; Writing – review & editing: All. M.M. is the guarantor of the study, has full access to all the data, and took responsibility for the integrity of the data and the accuracy of the data analysis.

### CONFLICT OF INTEREST

Malard reports honoraria from Sanofi, Amgen, Novartis, BMS, Astrazeneca, Therakos, Priothera, MSD, and Jazz Pharmaceuticals, all outside the submitted work.

Mohty reports grants and lecture honoraria from Janssen, Sanofi, MaaT Pharma, and Jazz Pharmaceuticals, lecture honoraria from Celgene, Amgen, BMS, Takeda, and Pfizer, and grants from Roche, all outside the submitted work.

The other authors declare no competing financial interests.

### ETHICS STATEMENT

The IACH Steering Committee (www.iach.org) approved the conduct of this survey in accordance with standard professional practices. The survey was not an interventional study and was conducted in accordance with the highest ethical standards.

### PATIENT CONSENT STATEMENT

This survey did not report any patient data. However, survey participants provided informed consent for publication of the survey results.

## Data Availability

Data sharing is available through the IACH office (info@clinical-hematology.org)
